# Investigation of environmental and health impacts solid waste management problems and associated factors in Asella town, Ethiopia

**DOI:** 10.1016/j.heliyon.2024.e28203

**Published:** 2024-03-17

**Authors:** Amde Eshete, Alemayehu Haddis, Embialle Mengistie

**Affiliations:** a Arsi University, College of Health Science, Public Health Department, Asella, Ethiopia; b Jimma University, College of Medicine and Public Health, Department of Environmental Science and Technology, Jimma, Ethiopia; c Hawassa University, College of Health Science, Department of Public Health, Hawassa, Ethiopia

**Keywords:** Health impact, Solid waste, Solid waste management, Ethiopia, And, Health intervention

## Abstract

Solid wastes are substances thrown away by the institutions of individual households in the urban community. The solid waste management practice in Asella town was inappropriate caused environmental pollution and exposed different urban health difficulties. The main objective was to determine the environmental and health impacts of solid waste management problems and associated factors in Asella town. Data collection was carried out through a house-to-house community survey process with a method of cross-sectional study design. A total sample size of 418 households was selected and implemented a simple random and systematic probability sampling technique. The statistical analysis of the binary logistic regression model was used to perform the correlational test about health issues in Asella town.

The environmental impact assessment indicators were 13.4% soil pollution, 31.6% air pollution, 20.8% environmental deterioration, and 34.2% water pollution. The health impact indicators were 49.5% respiratory diseases, 18.2% asthmatic (bronchitis) cases, 15.8% diarrheal diseases, 14.8% protozoan illnesses, and 1.7% cancer cases.

The odds ratio of females AOR = 1.18; 95%, CI:0.48–2.89, educational districts of can't read and write AOR = 2.31: 95% CI = 0.48–11.1, primary cycle AOR = 2.32, 95% CI:0.58–9.21, Secondary cycle AOR = 2.19, 95% CI = 0.60–7.98, and tertiary cycle AOR = 4.98, 95% C1.06–23.46. the odds ratio of house ownership of government AOR = 2.95, 95% CI:0.54–16.14, private households AOR = 4.18, 95% CI:0.79–22.16 and rented house property AOR = 1.71, 95% CI:0.32–9.18. The odds ratio of income group of lower status AOR = 2.0, 95% CI:0.91–4.98, middle income AOR = 5.7, 95% CI: 0.73–44.53 and higher income AOR = 2.8, 95% CI:0.35–23.14. The odds ratio for sorting of solid waste AOR = 1.38, 95% CI:0.56–3.40, and reusing of solid waste AOR = 7.90, 95% CI:2.12–29.42. Thus, the odds ratio of reusing solid waste was statistically significant correlated factors that limit health issues in Asella town.

Therefore, the inadequate solid waste management practice was a query for environmental and health impacts in Asella town. The principles of reusing, reducing, and recovering solid waste management practice must be supported by professional interventions and government policy.

## 1 Introduction

Solid waste is any material discarded as unwanted solid substances by the original owner of this property [1,2]. The adopted definition from the constitution of the federal democratic Republic of the Ethiopian government published on Negarit Gazeta's issue (2007:3525), stated that “solid waste” is anything discarded as unwanted substances neither a liquid nor a gas item [3]. Urbanization, industrialization, rising populations, and economic growth were some of the factors that contributed to an increase in the per capita generation rate of solid wastes. Managing solid waste is a challenging task across the world [4,5].

### 1.1 Characterization of solid wastes

Municipal solid waste (MSW), also known as garbage, trash, or refuse, is waste generated by the sources of residential (single and multifamily dwellings), commercial (bars, hotels, restaurants), institutional (offices, schools, prisons, hospitals, airports), and industrial (manufacturing, fabrication center). Municipal services (landscape maintenance, street sweeping), non-recycled building materials, and demolition garbage [6].

### 1.2 Solid waste management systems

Solid waste management (SWM) is a significant issue for society and administration, particularly in an urban environment struggling with rapid population expansion and continuously increasing volumes of solid waste [5,[7], [8], [9]]. The specific classification of solid waste management activity is composed of generation, storage, collection, transportation, treatment, and final disposal [10]. Financial problems in implementing effective and efficient solid waste management systems were a main challenge for many developing countries [11].

In Ethiopia, the activity of solid waste management systems was fixed to collection, transportation, and disposal to free space near urban/city environments. There were no properly constructed facilities such as communal garbage storage containers used for solid waste collection services in urban environments. In the majority of Ethiopian towns, 30%–50% of the solid waste generated was not collected by the municipality's solid waste administration office [12,13]. This scenario exposed urban residents to practices of open-field solid waste disposal mechanisms with the consequences of environmental pollution and the spread of communicable diseases [[14], [15], [16]].

### 1.3 Solid waste disposal facility

The main solid waste disposal facilities used especially in low- and middle-income developing countries in Latin America, Africa, and Asia towns were open-field disposal followed by dumping into rivers or streams even in roadside constructed channels diverted the flow of stormwater [4]. The magnitude of open-field solid waste disposal practice was 50%–80% in the towns of developing countries including Ethiopia [5]. The unscientific open-field disposal system of solid waste was exposed to different environmental pollution and affects the health of living people near surrounding dumpsites [17]. Thus, it causes water, soil, and air pollution directly affects human health by transmitting different communicable diseases [18,19].

### 1.4 Environmental and health impacts of solid waste management problems

The inadequate solid waste management systems were a victim of a threat to the person's quality of life. The relationship between the environment and health is extremely crucial to public health [20]. To protect and promote children's health and the welfare of the community, a safe and healthy environment is required. Globally ensuring environmental sustainability has been given priority attention by implementing solid waste management systems which was stated in United Nations Sustainable Development Goals 11.5 [5]. The consequences of poor solid waste management practices cause environmental pollution and affect human health [21].The vast majority of the global burden of communicable disease transmission was caused by environmental pollution. The morbidity and mortality rate of respiratory infections, diarrheal diseases, and vector-borne diseases reached 24% of all global deaths and 28% of deaths among children as a consequence of environmental pollution specifically with water, soil, and air pollution [[22], [23], [24]].

An aesthetic problem occurred because of poor solid waste handling practices contributing to pollution of soil, and air, and direct or indirect contamination of sources of water (surface and groundwater) which deteriorated environmental sanitation. This situation causes a variety of health effects such as gastrointestinal sickness, respiratory illnesses, dermatological infection, genetic damage, eye, and nose irritations, and psychological disorders [5,11,25,26].

Birth defects, cancer, congenital disorders, and preterm births were other health problems caused by the contamination of toxic chemicals [27] emitted from solid waste discarded environment. The decomposition of organic solid wastes produces gases of ammonia, and hydrogen sulfide including bad odors which aggravate allergic reactions in the anatomical organs of the nose, throat, and eyes [8,28].

The composted solid waste can create an excellent breeding habitat for rodents and other medically important arthropods, Because of the infestation of insects and rodents, urban dwellers were vulnerable to infection and the spread of vector- and rodent-borne diseases [29,30].

Children, pregnant women, older age groups, and waste scavengers were the most vulnerable groups for disease transmission as a result of environmental pollution. Solid waste dump sites served as breeding grounds for flies, rodents, and other carnivore animals such as dogs, cats, and hyenas, which were responsible for the transmission of many communicable diseases [2,11,31]. The purpose of this study was to detect the prevalence and correlating solid waste management systems applied as a solution of impact reduction on the environment and health.

Therefore, the general objective was to determine the environmental and health impacts of solid waste management problems and associated factors in Asella town, Ethiopia.

## 2 Methods and materials

### 2.1 Study area

**Fig. 1:**
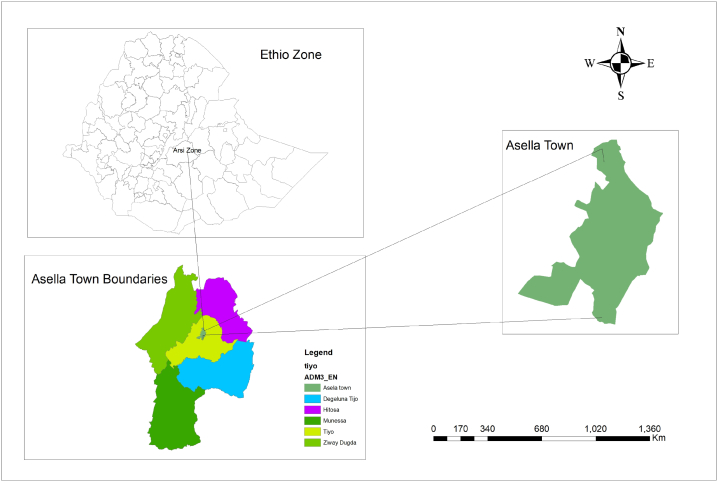
Map of Asella town, Arsi zone, Oromia regional state, Ethiopia.

Asella, a town in the Arsi zone, is located at a distance of 175 km from Ethiopia's capital city of Addis Ababa, which serves as the zonal administrative center. The town is located at latitude 7° 57′ N, longitude 39° 7′ E, and an altitude of 2431 m above sea level [32]. [33] According to census data, 68,269 people were living in Asella town. Among them, 33,826 were men and 34, 443 women. The geographic boundary map in Ethiopia indicates the Arsi zone site of Asella town as shown in Fig. 1.

### 2.2 Study design

During the assessment of the house-to-house community survey process, a cross-sectional study design was used in each kebele of Asella, the smallest town administrative unit.

### 2.3 Study variables

#### 2.3.1 Dependent variables

Health impacts.

#### 2.3.2 Independent variables

Gender, education (can't read and write, primary cycle, secondary cycle, and tertiary cycle), income (lower income, middle income & higher income), house ownership (government, private and rented houses), sorting, and reusing practice of solid wastes.

### 2.4 Sample size

The actual sample size for conducting the community survey process was determined by using the single proportion calculation. The investigator calculated the maximum number of representative sample sizes with a 50% simulation proportion. Then, the Cochrane formula was used to calculate the calculated sample size.

n=(z∝)/2〗EXP2×(p×q)/∝EXP2=(1.96)EXP2×0.5×0.5/〖0.05〗EXP2=384

Where n_o_ = sample size; p = proportion where p = 0.5; d = precision = 0.05; zα/2 = 1.96.

By adding 10% for the non-response rate, the final sample size was 423 households.

### 2.5 Data collection process

The smallest administrative division of a town which was known as a kebele set up to collect data from individual households. Totally eight kebele combined to make up Asella town. The sample size was proportionally distributed based on the total number of houses in each kebeles of Asella town. The random sampling procedures were equally done among all kebeles of Asella town. The determination of K^th^ value was calculated as each kebeles total houses divided by the sample size i.e. 422 for implementing a systematic data collection process. The intervals were 4–6 homes among the neighboring households in the study sites. The reason for these variations was that the total number of houses in each kebele was not the same numbers. The proportion for each kebeles was calculated as the total number of houses within the kebeles divided by the total number of residential houses in Asella town. Finally, each proportion was multiplied by sample size i.e.422 given the total number of houses selected as representative sample houses within the target kebeles (Table 1). The data collection process continued with the directions of roadsides until the end of each home's information-gathering process. The collected data were finally entered into data entry software.

**Table 1:** The sample house distribution for the data collection process in Asella town.

S/No	Kebele	Total residential Houses per Kebeles	Proportion each kebele residential houses per Total Asella town residential houses	Representative total sample houses Per kebeles	kth value per kebeles
1.	Anko	2119	0.134	57	6
2.	Burkitu	2467	0.156	66	6
3.	Buseta	1771	0.112	47	4
4.	Chilalo	2277	0.144	60	5
5.	Combolcha	1484	0.094	40	4
6.	Halila	1550	0.098	41	4
7.	Hundegudina	2530	0.16	68	6
8.	Wolkesa	1613	0.102	43	4
Total		15,811	1.007	422	

### 2.6 Data collection tools (personnel, instruments, and measurements)

The investigator trained the field supervisors and data collectors about the objectives of the anticipated research project. Before beginning the fieldwork, the researcher thoroughly explained and discussed the data collection tools of the prepared checklists and questionnaires. Health professionals who worked in different governmental health facilities of Asella town were selected for data collectors.

Checklists and open and closed-ended questionnaires were prepared as data collection tools. For precision and consistency, these tools had first been developed in the English language, then translated into the Afan Oromo language, and finally retranslated back into the Amharic language. All the information necessary for this study was included in the prepared checklists and questionnaires.

### 2.7 Data processing and analysis

The statistical analysis and interpretation were performed after the collected data was coded, entered into EPI Data 4.6 software, and exported to Minitab 21.2 statistical analysis software. The significant variables were tested by models of binary logistic regression techniques. The tested variables were Gender, education (can't read and write, primary cycle, secondary cycle, and tertiary cycle), income (lower income, middle income & higher income), house ownership (government, private, and rented houses), sorting, reusing practice of solid wastes.

### 2.8 Ethical clearance

Ethical clearance was obtained from the Jimma University Ph.D. Research Directorate office after getting the approval of the ethical clearance committee to take this investigation in Asella town.

## 3 Results

### 3.1 Sociodemographic features

The survey assessment on solid waste management systems during the community study had a response rate of 98.8%. This shows the majority of respondents were positive for the surveys on the health and environmental impacts of solid waste management problems in Asella town. Among the respondents 1.2% did not provide the requested information while they were not found in their homes during the data collection period because of their problems Among the interviewers, 281 (67.2%) were female and 137 (32.8%) were male. In developing countries like Ethiopia, females were more focal person than males and usually carried residential environments. The mean age of the respondents was 39.1 years, with a 95 percent confidence interval of 38–40 years. The adults can provide better responses to the questions so that the survey data was tainted confidential information for the analysis process.

Among the respondents, 396 (94.7%) were well known about the health problems caused by the inadequate solid waste management system though the rest 22 (5.3%) were unaware of it. The educational status assessment was 17.7% female and 9.8% male clustered in the tertiary cycle, and 20.8% females and 8.4% male in the second cycle well-known about the health and environmental effects of poor solid waste management practices in Asella town (Table 2).

**Table 2:** Assessment of the educational status of respondents on the gender interviewers of solid waste management system in Asella town.

Education	Gender		
	Female	Male	Total
Can't read & write	42	13	55
Percent response (%)	10	3.1	13.2
Others	20	14	34
Percent response (%)	4.8	3.3	8.1
Primary cycle	58	34	92
Percent response (%)	13.9	8.1	22
Secondary cycle	87	35	122
Percent response (%)	20.8	8.4	29.2
Tertiary cycle	74	41	115
Percent response (%)	17.7	9.8	27.5
All	281	137	418
Total percent response (%)	67.2	32.8	100

### 3.2 Composition of solid waste

**Fig. 2:**
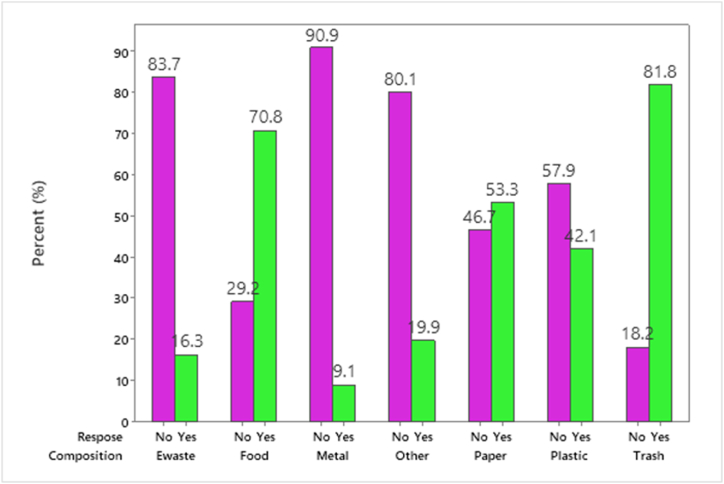
The characterization of solid waste composition in Asella town, Ethiopia.

The result of solid waste characterization was 81.8% trash, 70.8% food waste, and 9.1% metallic waste. The prevalence of solid waste composition was indicated in the bar graph Fig. 2. The result of the community survey was 32.3% organic solid waste whereas 9.6% inorganic solid waste was produced from the households of Asella town, Among the respondents 41.9% classify solid wastes into specific collection services however 58.1% disposed of as mixed solid waste at household storage equipment Fig. 2.

### 3.3 Solid waste management practice

In the sorting of solid waste handling practice was 41.1% of households applied into characterized composition classes although the remaining 58.9% unable to procedure subsequently. 119 (28.5%) women and 53 (12.7%) men were cooperative in the solid waste sorting process in their homes (Table 3).

**Table 3:** Analysis of the solid waste sorting process relating to gender in Asella town.

Sorting solid waste	Gender		
	Female	Male	Total
Yes	119	53	172
Percent response (%)	28.5	12.7	41.1
No	162	84	246
Percent response (%)	38.8	20.1	58.9
Total	281	137	418
Percent response (%)	67.2	32.8	100

**Fig. 3:**
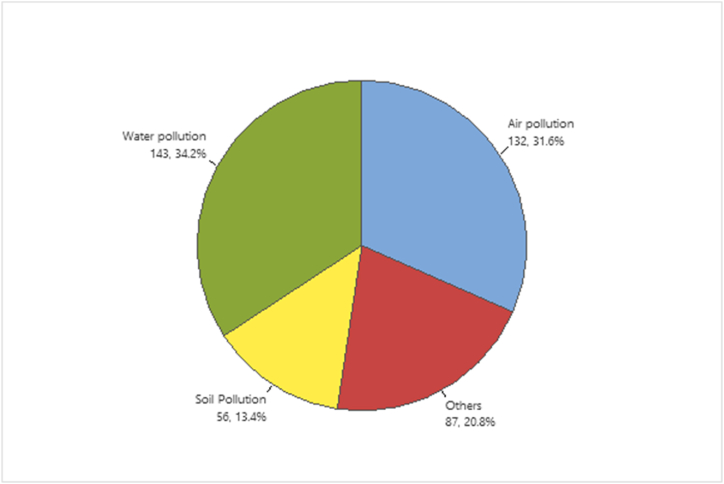
The environmental impact indicators of improper solid waste management system in Asella, Ethiopia.

The assessment of environmental factors of Asella town solid waste management problems obtained significant consequences. The environmental impact indicators were 143 (34.2%) water pollution, 132 (31.6%) air pollution, 56 (13.4%) soil contamination, and 20% various environmental difficulties Fig. 3. The result of solid waste management practice in Asella town was poor and highly exaggerated environmental pollution.

The health impact indicators were 207 (49.5%) respiratory disorders, 76 (18.2%) asthma cases, 66 (15.8%) diarrheal diseases, 62 (14.8%) parasite infections, and 7 (1.7%) cancer episodes. The consequence of the solid waste management systems in Asella town was so critical that needs to change to become a major public and government concern of the urban population in Asella, Ethiopia.

The survey revealed 14.6% mosquitoes, 18.4% rats, 8.9% carnivore animals, and 42.6% houseflies retained in a favorable of arthropods and carnivores’ animals with solid waste dump sites. The outcomes of these insects and animals are highly under the question of health issues, especially for people living near solid waste dump sites in the case of Asella town.

### 3.4 Correlation factors for assessment of health impacts

The odds ratio of females (AOR = 1.18; 95%, CI:0.48–2.89), educational clusters of can't read and write (AOR = 2.31: 95% CI = 0.48–11.1), primary cycle (AOR = 2.32, 95% CI:0.58–9.21), Secondary cycle (AOR = 2.19, 95% CI = 0.60–7.98), and tertiary cycle (AOR = 4.98, 95% C1.06–23.46). The odds ratio for government house ownership (AOR = 2.95, 95% CI:0.54–16.14), private house ownership (AOR = 4.18, 95% CI:0.79–22.16), and rented house ownership (AOR = 1.71, 95% CI:0.32–9.18). The odds ratio for lower income (AOR = 2.0, 95% CI:0.91–4.98), middle income (AOR = 5.7, 95% CI: 0.73–44.53) and higher income (AOR = 2.8, 95% CI:0.35–23.14). The odds ratio for sorting solid waste (AOR = 1.38, 95% CI:0.56–3.40) and reusing solid waste (AOR = 7.90, 95% CI:2.12–29.42). Among the investigated odds ratios reusing solid waste was a statistically significant issues that indicate the correlations of factors that limit health issues in Asella town.

## 4 Discussion

### 4.1 Environmental impacts

The rise in living standards and the continued expansion of the world economy led to resource depletion and an increase in solid waste generation rates. The inappropriate management of solid waste sorting, handling, collecting, transporting, and disposing activities can purposely affect the environment and human health. Therefore, each urban community member is important to practice efficient waste management systems with affordable and environmentally accountable technology [34].

The educational status of the higher class was more knowledgeable than lower education assemblages of solid waste management systems [35,36]. Similarly, current project outcomes exhibited a significant association between solid waste management systems and education status [37]. The results of this study may also support the aforementioned evidence given that higher educational status had developed better skills than lower educational groups Table 1.

After being collected from each residential household the solid waste was eventually transported to open-field disposal sites in Asella town. This was the oldest method of application of earlier handling of solid waste. The expected solid waste management system was practiced, kept, and arranged for mixed solid waste removal from housing environments. Evidence on open-field dumping of solid waste was exposed to fires, mechanical and physical hazards in aggregation with environmental pollution and health hazards. The magnitude of open field practice was 30–50% solid waste produced from undeveloped towns handled on streets and open areas pretentiousness key environmental and health risk [38].

The composition of solid waste was 9.8% plastic, 9.6% paper, and 62.1% food waste detected in a systematic review in Ghana [39]. The prevalence of food waste generation was high in Asella town compared to Ghana, the reason might be differences in sample sizes compatibly the variation in consumption type of food staff. It was crucial to solve the management of food waste problems to control their negative impacts on human health, even though the practice of characterizing the content of solid waste varied from country to country.

The use of technology to change ecosystems is one specimen of human activity that yields solid waste and may contaminate the environment. Air pollution and water pollution are the two types of pollution that are most prevalent in low-income countries. This contrasts with economies that are expanding quickly, back when chemicals and pesticides were the main causes of environmental deterioration due to their toxicity [40]. Environmental contamination was suitable for the growth and multiplication of microorganisms harmful to people's health as a source of communicable diseases [41].

The inadequate solid waste treatment systems were a reason for the pollution of air, land, and water. Therefore, deprived solid waste management schemes spoiled the ecosystem and led to chemical poisoning and microbial transmission [28,42,43]. Asella town practiced a similar circumstance.

The collected solid waste created a breeding ground for rodents, insects, and other animals with blackening water flow channels [44]. Similar insect and vermin infestations, as well as certain environmental concerns, also occur in the town of Acela. It is well-known arthropods and rodents affected the spread of many contagious diseases and they affected public health [5]. The larval stages of disease-carrying vectors like rats, flies, mosquitoes, and houseflies can be found in open organic waste dumps sites. Among the associated vector-borne diseases, dengue fever, malaria fever, and Zika virus were a few examples. In addition, there was a chance of getting leptospirosis, intestinal worms, diarrhea, and other water-borne diseases like hepatitis [5].

### 4.2 Health impacts

The current result supported the idea that most respondents were aware of the consequences of health problems caused by inappropriate solid waste management practices. These results were validated by research demonstrating on harmful impacts of inappropriate solid waste management on the environment and human health [45,46]. The study done in Mumbai made 81% of respondents aware of the link between poor solid waste management practices and health issues [46]. The difference with the current work might be the outcome of a behavioral change in the town of Asella by providing health education offered through the relevant healthcare professionals.

The spread of diseases like cholera and typhoid which are both typically understood as waterborne infections and other health issues has long been related to water pollution. Environmental pollution can result in non-communicable diseases like cancer, asthma, and several birth defects in addition to infectious diseases. The majority of the harmful effects of environmental pollution on health-related outcomes have been found in low-income nations, where it is thought that this form of pollution accounts for 90% of all fatalities [47].

The study conducted in the Yeka sub-city of ADDIS ABBA, and Ginchi town of Ethiopia was cholera, cough, diarrhea, and nose and eye irritations occurred because of environmental pollution [8,48]. Similarly, in Chiro Town environmental pollution and health risks such as diarrhea, pneumonia, typhoid fever, cough, and malaria occurred due to poor solid waste management problems [[49], [50], [51]]. Some of these contagious diseases were discovered in Asella town.

The investigation carried out in Malaysia showed that 75% of cases developed respiratory problems as a victim of poor solid waste management practices [28]. The fact of respiratory cases detected in Asella town even though the magnitude was low may be influenced by the difference in the virulence factors, natural immunity, and case detection rates.

The organic solid waste composition of Chiro was 90.4% [16] and more than 60% of the solid waste was produced in Hawassa City town [15]. The current result of organic solid waste in Asella town was lower than those from these stated towns. This variation might be because the characterization of solid waste in Asella town was low. Among the respondents, only 41.1% of households characterize their solid wastes but the rest 58.9% did not practice it properly. When decomposing organic solid wastes of aerobic and anaerobic microorganisms such as bacteria, fungi, protozoa, and algae must be under control to stop disease transmission cycles [37]. The mechanism of fico-aural disease transmission occurs since the decomposition process generates extra microbial contamination scenarios that lead to the communicability of illnesses through contaminated food, water, and soil. Additionally, the decomposition of organic waste produced an unpleasant odor an indication of air pollution that might create respiratory problems in nearby residents partly a health indicator of Asella town.

Sorting solid waste is a fundamental method for easing a community's burden on the environment and its residents' health. In Asella town, it was partially practiced. In a similar study performed in Iran, it was shown that 50% of women and 37.6% of men participated in the sorting of solid waste [35]. Thus, Unsorted solid waste poses serious concerns to public health and environmental issues since it might be dumped in the nearby environment as mixed or commingled solid waste. In comparison to Iran, Asella had lower participation in the separation of solid waste. This may be spurred by the difference in the respondents' levels of knowledge in Iran and Asella. Overall, both findings displayed that women were a better target for sorting solid waste than men (Table 2).

The AORs ratio of binary logistic regression results supported significant statistical evidence indicating that exposure to inadequate solid waste management practices worsens environmental pollution further donated to health questions.

## 5 Conclusion

Solid waste is a material thrown away or eliminated or discarded items yet out of liquid or gaseous substances generated by people living in individual households in the case of Asella town. The prevalence of generating items composition was 9.6% inorganic substances and 32.2% organic solid wastes produced from residential environments in Asella town.

The survey outcomes of water pollution, air pollution, soil pollution, and other environmental failures were 34.2 %, 31.6 %, 13.4 %, and 20.8 % were sensor indicators of environmental impact determinants in Asella town.

The surveyed health impact assessment indicators were parasite infections (14.8%), diarrheal diseases (15.8%), respiratory diseases (49.5%), asthma (bronchitis) cases (19.2%), and cancer victims (1.7%) because of poor solid waste management systems of Asella town.

The discoveries of 14.6% mosquitoes, 18.4% rats, 8.9% carnivore animals, and 42.6% houseflies persisted positively exciting arthropods and carnivores’ animals through solid waste dump sites. Thus, insects and animals are highly under question for health issues particularly people living neighboring solid waste dump sites in Asella town.

The results showed that 41% of households handle sorting of solid wastes among this, 28.5% of the activities were carried out by female partners while 12.7% by respective male gender groups. So, in Asella town females were the responsible persons for sorting solid waste due to house cleaning activities carried by females than male gender groups.

The odds ratio female AOR = 1.18; 95%, CI:0.48–2.89, can't read and write AOR = 2.31: 95% CI = 0.48–11.1, primary cycle AOR = 2.32, 95% CI:0.58–9.21, Secondary cycle AOR = 2.19, 95% CI = 0.60–7.98, tertiary cycle AOR = 4.98, 95% C1.06–23.46. The odds ratio of government house ownership AOR = 2.95, 95% CI:0.54–16.14, private house ownership AOR = 4.18, 95% CI:0.79–22.16 and rented house ownership AOR = 1.71, 95% CI:0.32–9.18. the odds ratio of lower income AOR = 2.0, 95% CI:0.91–4.98, middle income AOR = 5.7, 95% CI: 0.73–44.53 and higher income AOR = 2.8, 95% CI:0.35–23.14. the odds ratio for sorting solid waste AOR = 1.38, 95% CI:0.56–3.40, and the associated significant odds ratio of reusing solid waste AOR = 7.90, 95% CI:2.12–29.42 were detected about health issues by using binary logistic regression models.

Therefore, the overall solid waste management practice was inadequate so a major concern of shifting environmental and health impact influencing systems in Asella town.

## 7 Recommendation

The solid waste management systems of Asella town have to be changed to enhance environmental and health problems which are the targets of sustainable development goals.

The generated solid waste by each household needs to be picked up by the municipality, a private individual, or other solid waste collectors at least once a week.

The open-field dumping of solid waste must be avoided from urban solid waste disposal facilities.

The principles of reusing, reducing, and recovering solid waste management practice must be aided by professional skills and government policy.

The determinations of the association between solid waste management systems concerning the composition of solid wastes further outlined by future researchers.

The magnitude of health impacts with specific environmental pollution because of unscientific solid waste management systems suggested for future researchers, especially in developing countries.

Further studies also suggested the complications of global environmental burdens of greenhouse effects for current advanced environmental pollution of urban solid waste management problems.

## Ethics approval and consent to participate

Ethical clearance was approved by the Jimma University Ph.D. research directorate office. The investigator declared that oral consent was obtained from households while collecting data from sampled households in Asella town. The oral consent that was obtained from the respondent was “I fully agree to provide you the exciting information helpful for your research work”.

## Funding

10.13039/501100005068Jimma University had supported financial supply for data collectors peridium and transport cost coverage services.

## Availability of data and material

Data was presented by tables and graphs throughout the manuscript. If requested it is available with the corresponding author.

## CRediT authorship contribution statement

**Amde Eshete:** Writing – review & editing, Investigation. **Alemayehu Haddis:** Project administration. **Embialle Mengistie:** Conceptualization.

## Declaration of competing interest

The authors declare that they have no known competing financial interests or personal relationships that could have appeared to influence the work reported in this paper.
